# Supplementary material for: Reciprocating RNA Polymerase batters through roadblocks
Source: Nat Commun. 2024 Apr 12;15:3193. doi: 10.1038/s41467-024-47531-x (PMC11014978; doi:10.1038/s41467-024-47531-x)
Supplement: Supplementary file 1 — Supplementary Information [file 41467_2024_47531_MOESM1_ESM.pdf]

Reciprocating RNA Polymerase Batters Through Roadblocks

Supplementary Information

Supplementary Figures 1-11

Supplementary Table 1

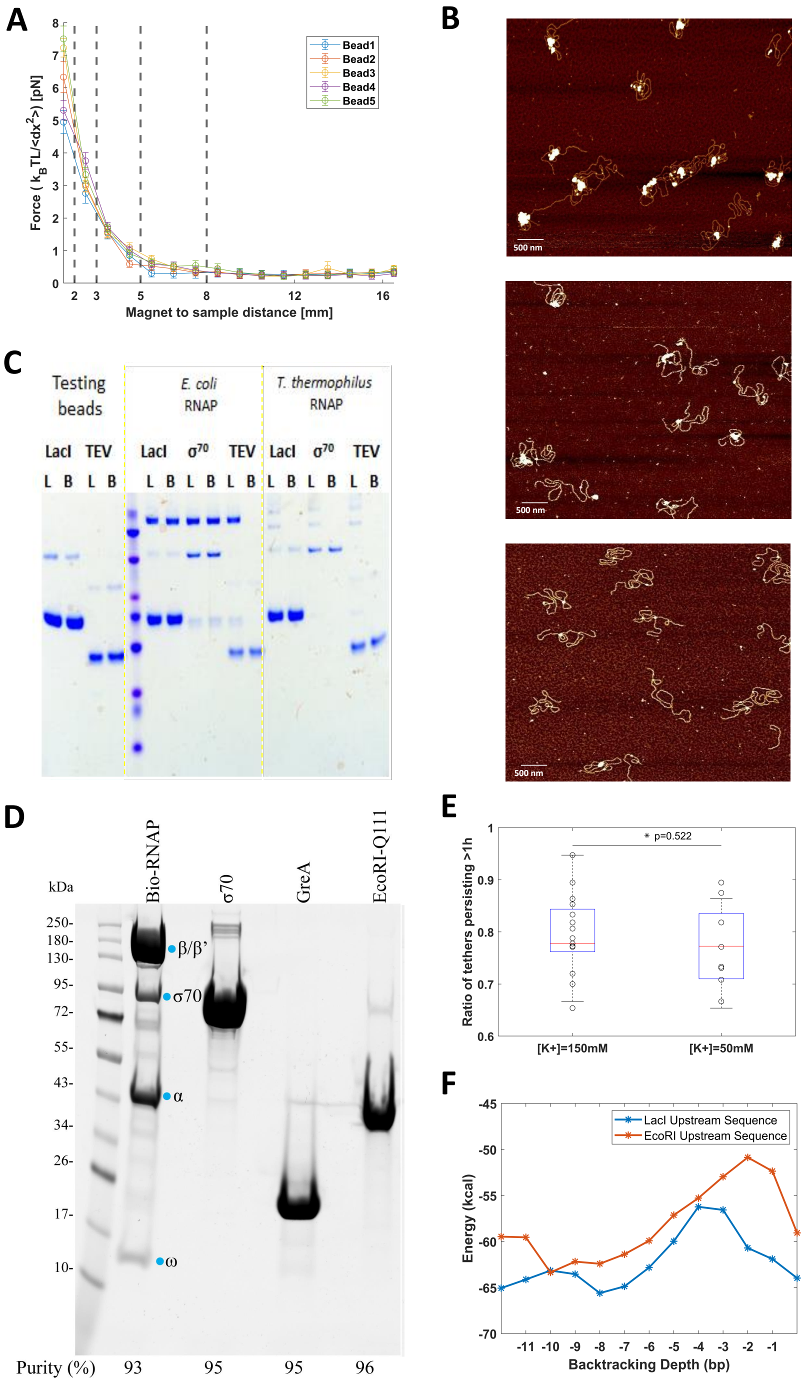

A
Force ($k_BTL/<dx^2>$) [pN]
Magnet to sample distance [mm]
Bead1
Bead2
Bead3
Bead4
Bead5
B
500 nm
C
Testing beads
E. coli RNAP
T. thermophilus RNAP
LacI TEV LacI σ70 TEV LacI σ70 TEV
L B L B L B L B L B L B L B L B
D
kDa
Bio-RNAP
σ70
GreA
EcoRI-Q111
250-
180-
130-
95-
72-
55-
43-
34-
26-
17-
10-
β/β'
σ70
α
ω
Purity (%) 93 95 95 96
E
Ratio of tethers persisting >1h
* p=0.522
[K+]=150mM
[K+]=50mM
F
Energy (kcal)
Backtracking Depth (bp)
LacI Upstream Sequence
EcoRI Upstream Sequence

Supplementary Figure 1: Verification of protein preparation, binding activity, tether integrity, RNAP polymerase activity, and estimates of backtracking efficiency.
(**A**) Force varies among beads. Force vs. magnet-sample distance is shown for five different beads. The dashed lines indicate the magnet-sample distances used in experiments corresponding to 5, 2, 0.7 and 0.2 pN, respectively. Since different beads may produce forces of slightly different magnitudes, the rescaling routine discussed in Fig. 2 was applied to normalize the tether lengths.
(**B**) AFM images showing EcoRI Q111 protein bound to DNA at different concentrations. (**top**) EcoRI Q111 proteins aggregated and frequently bound non-specifically at 150 nM. (**middle**) EcoRI Q111 proteins were more specifically bound and formed fewer aggregates at 50 nM. (**bottom**) EcoRI Q111 proteins bound specifically to DNA at 25 nM, but failed to saturate the binding sites of all templates. Considering these results, 45 nM EcoRI Q111 was used in MT assays to achieve a high and low levels of specific and non-specific binding respectively.
(**C**) Co-immunoseparation of LacI with RNAP. (**left**) His-tagged LacI or tobacco etch virus (TEV) protease efficiently partitioned with Talon Dynabeads; L, Loaded; B, bound. (**middle**) Untagged RNAP incubated with His-tagged LacI or $\sigma 70$, but not TEV, efficiently co-partitioned with Talon Dynabeads. (**right**) *T. thermophilus* RNAP incubated with His-tagged LacI, $\sigma 70$, or TEV did not co-partition with Talon Dynabeads.
(**D**) A densitometric analysis of an SDS-PAGE separation of purified proteins reveals the purity of the RNAP, GreA, and EcoRI Q111 used in this study.
(**E**) Tethers are robust versus salt concentrations. Each data point indicates the ratio of the number of tethers that endured more than one hour of observation to the total number of tethers within a micro-chamber. Measurements were conducted in the absence of NTPs and roadblock protein.
(**F**) The free energy of ECs was calculated during backtracking upstream of LacI and EcoRI Q111 roadblocks as previously described [1]. ECs are less stable upstream of the EcoRI roadblock, which might suggest faster backtracking-recovery cycles.

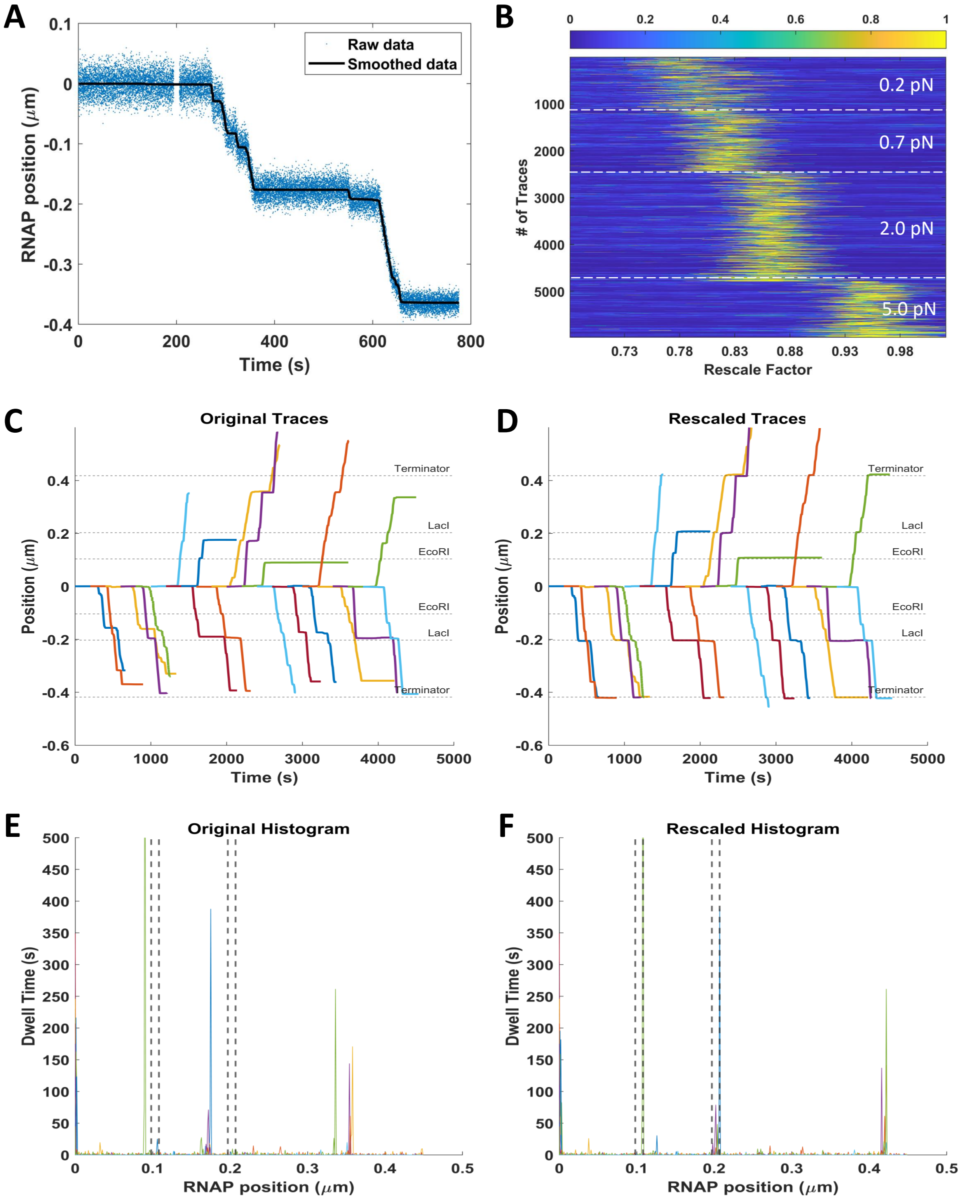
A
Raw data
Smoothed data
RNAP position (μm)
Time (s)
B
# of Traces
0.2 pN
0.7 pN
2.0 pN
5.0 pN
Rescale Factor
C
Original Traces
D
Rescaled Traces
Terminator
LacI
EcoRI
Position (μm)
Time (s)
E
Original Histogram
F
Rescaled Histogram
Dwell Time (s)
RNAP position (μm)

Supplementary Figure 2: **Representative transcription traces and processing for analysis**

(**A**) An example transcription record under -2 pN (opposing) force and the corresponding smoothed monotonic trace. The time series during NTP injection (blank area at ∼200 seconds in raw data) were manually excluded from the analysis. The smoothed time series (black line) was inferred from the raw time series using the "Total Variation Denoising Algorithm" [2]. For further analysis, the smoothed time series were resampled at 50 Hz interpolating the missing values using the MATLAB "interpl1" 1D-interpolation algorithm.

(**B**) Summary of the normalized similarity coefficients between the raw and rescaled traces during the rescaling process. A model histogram **X** was defined with 1 at roadblock sites (0.203 µm for LacI roadblock and 0.104 µm for EcoRI roadblock ± 10 nm, using 1 nm = 0.34 bp) and terminator sites (0.418 µm ± 10 nm) and 0 at other positions. The lengths in each time series, were multiplied by linearly spaced factors ranging from 0.7 to 1.03 with 0.001 increment to generate a group of rescaled time series and produce dwell time vs. RNAP position histograms. Then a coefficient, S, of similarity of each rescaled histogram to the model histogram **X** was calculated, $S = \sum X_i * x_i$. The rescaling factors that produced the highest similarity coefficients were selected for each trace (as shown in panels **C**- **F**). Traces for tethers under the same force condition produced similar rescaling factors. Greater tensions stretched DNA tethers further, and therefore produced rescaling factors closer to 1.

(**C**) A collection of 15 representative, smoothed transcription records and (**E**) overlaid histograms of the dwell times exhibit pauses dispersed around the promoter, roadblock and terminator positions before rescaling. The starting time of different records were shifted to avoid overlap.

(**D**) The same collection of transcription records in (**C**) are shown after rescaling to remove the effects of force variations that alter extension. (**F**) The overlaid histogram of dwell times for the rescaled transcription records shows peaks (higher probability of pausing) at promoter, operator, and terminator sites. Any pauses greater than 20 seconds within the expected roadblock positions ± 10 nm (intervals marked by dashed vertical lines) were identified as roadblock-induced pauses. Traces for which the tether length did not change after RNAP reached the roadblock site + 10 nm were identified as indefinite pauses.

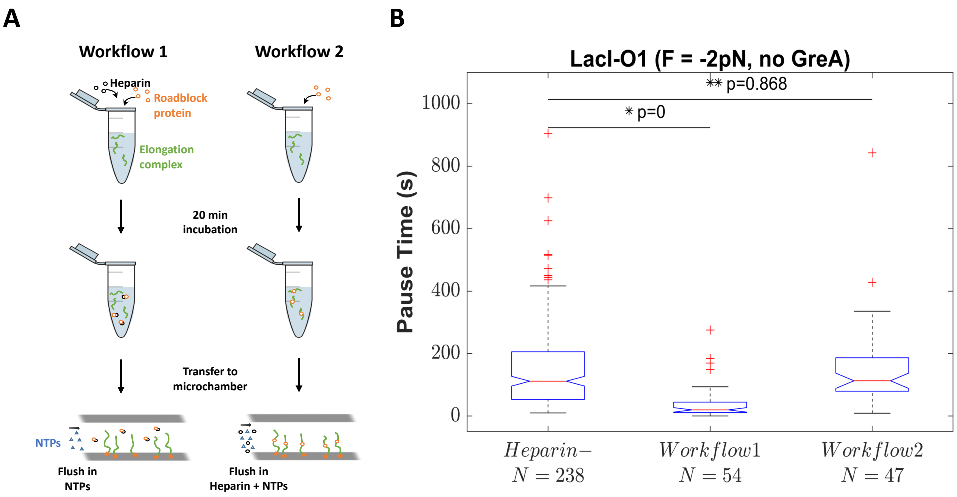

Supplementary Figure 3: **Experiments with heparin suggest roadblock rebinding negligibly affects pause times.** (**A**) Experiments with heparin were performed to determine whether roadblock proteins might dissociate but rapidly re-associate at DNA binding sites to extend pauses. (Left) In Workflow 1 (positive control), heparin at a concentration of 10 µg/mL was introduced with LacI to diminish roadblock formation before adding NTPs to initiate transcription. (Right) In Workflow 2, after incubation to produce LacI roadblocks on stalled EC-DNA templates, the same concentration of heparin was introduced with NTPs to sequester LacI dissociating during transcription. (**B**) Shorter pauses measured following workflow 1 (middle column) versus no heparin (left column) indicate that heparin diminished the formation of LacI-DNA roadblocks. Using the same heparin concentration to sequester LacI dissociating during transcription did not significantly shorten pauses at roadblocks formed via workflow 2. This indicates that re-association of LacI at roadblock sites did not influence pause measurements.

Supplementary Figures 4-7: Determining pause lifetimes from experimental CCDFs.

The roadblock-induced pause times were determined for all traces in each condition, and the complementary cumulative distribution functions were plotted. Since pause times shorter than 20 seconds could not be differentiated from ubiquitous pauses and were excluded in the pause time detection step, the CCDFs were fitted to the shifted exponential function $y = 1 - \mathrm{EXP}(-(x - 20)/\tau)$. The lifetimes ± 90% confidence intervals are shown in Supplementary Figure 4–7. Single exponential fits yielded a $R^2 > 0.95$ for all CCDFs, except for transcription traces in the presence of LacI-O2 under opposing force and without GreA. The p values of two-sided two-sample t-tests are included.

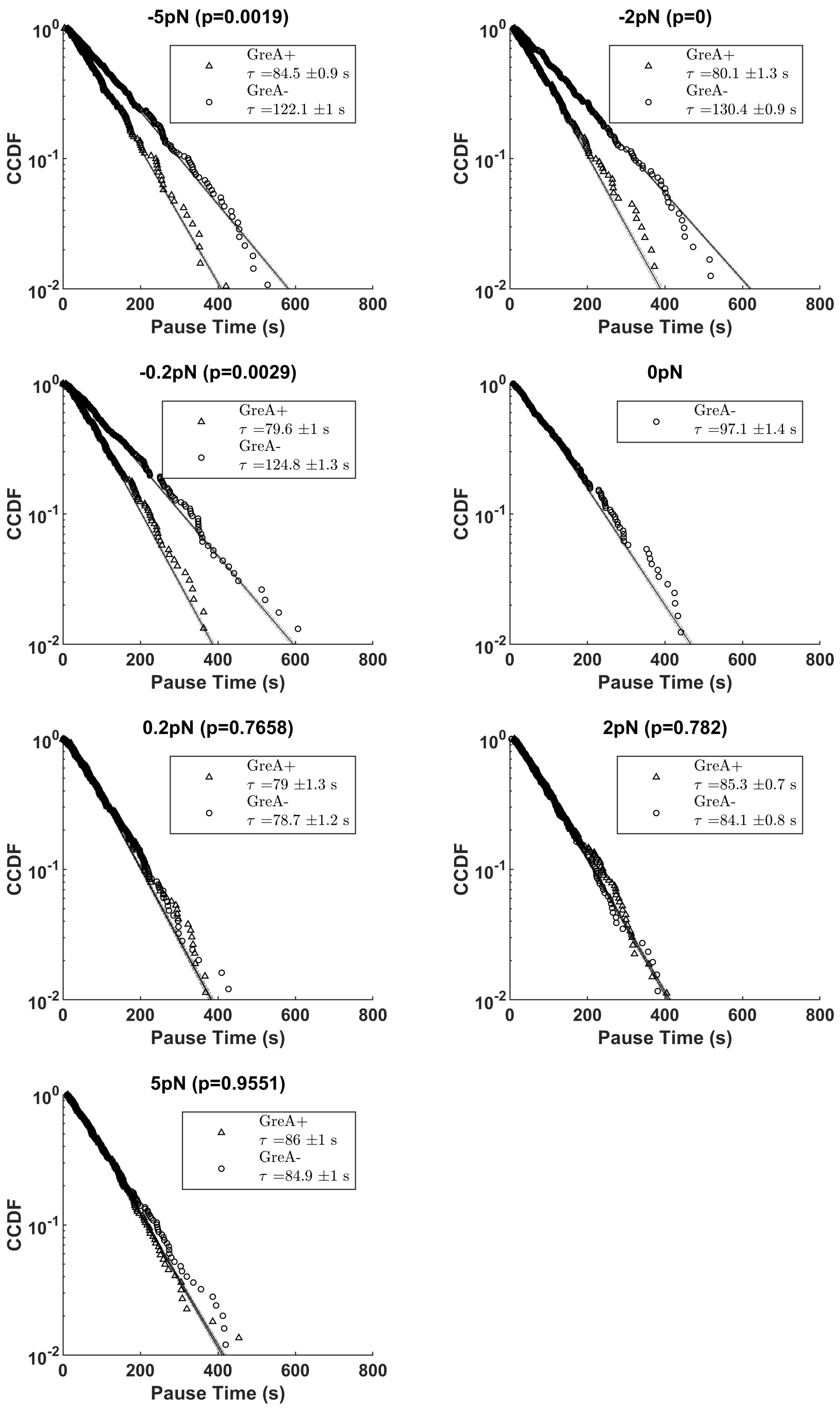

Supplementary Figure 4: LacI-O1 pause time CCDFs and characteristic lifetimes from exponential fittings.

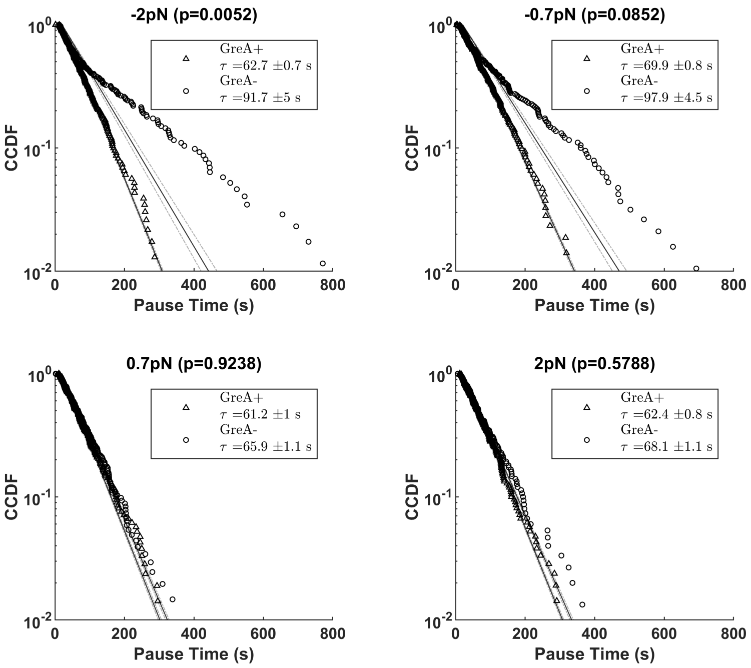

Supplementary Figure 5: LacI-O2 pause time CCDFs and characteristic lifetimes from exponential fittings.

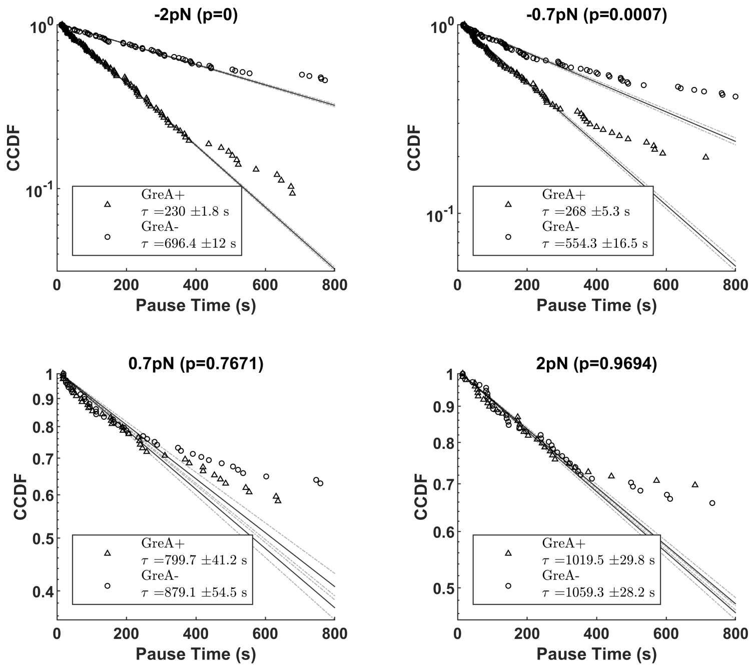

Supplementary Figure 6: LacI-Os pause time CCDFs and characteristic lifetimes from exponential fittings.

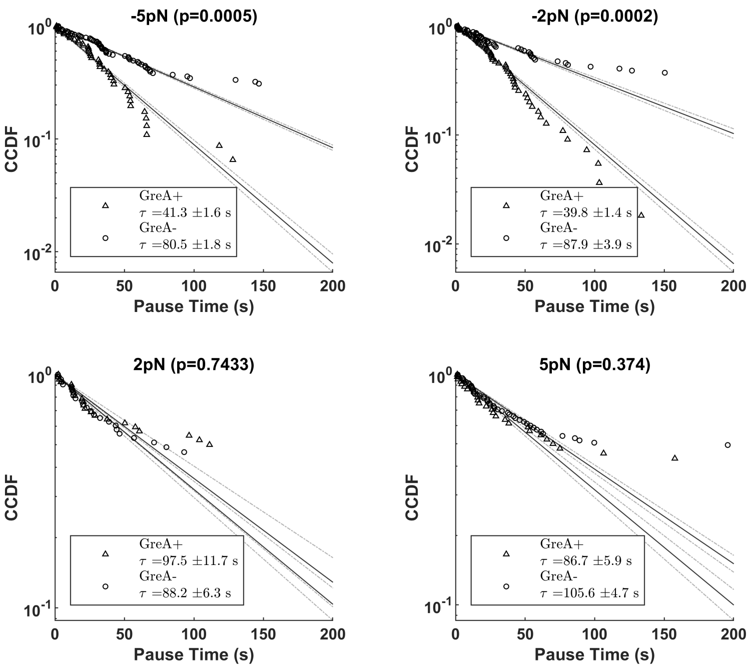

Supplementary Figure 7: EcoRI Q111 pause time CCDFs and characteristic lifetimes from exponential fittings.

Supplementary Figures 8-9: Fitting model parameters using experimental CCDFs.

To fit model parameters, we created a routine that accepts values of k_1, k_2, k_3 and P_1 to simulate 1000 pause times using Algorithm 1 of the main text to generate a pause time CCDF. Since the experimental data included only pause times greater than 20 seconds, the routine repeats Algorithm 1 to accumulate 1000 pause times longer than 20 seconds. Then, we used the MATLAB Surrogate Search global optimization algorithm to search for values of parameters that minimize the R^2 between the simulated and experimental CCDFs. Since GreA had little effect on pause lifetimes under assisting force, we postulated that RNAP transit under assisting force occurs through a passive pathway, $k_{\text{passive}} = k_3$. Therefore, we fit k_3 of roadblocks under assisting force conditions using the corresponding pause time distributions without considering the active pathway parameters k_1, k_2 and P_1. The values of k_1, k_2, and P_1 were set to zero, effectively excluding the active pathway from the model. The optimized k_3 values yielded CCDFs similar to experimental CCDFs ($R^2 > 0.98$) as shown in Supplementary Figure 8. With k_3 values set, values of k_1, k_2, and P_1 were fit using the experimental CCDF under opposing forces with/without GreA conditions. For GreA+ conditions, backtrack recovery was accelerated and k_2 was set to infinity. At all types of LacI roadblocks, RNAP should have same backtracking and recovery rates k_1 and k_2, since LacI binding sites have the same upstream DNA sequence. The EcoRI Q111 binding site was embedded in a different DNA sequence and might have different k_1 and k_2 values. P_1 should relate to the strength of roadblocks and was assumed to be linearly dependent on k_3. Overall, we fit a set of k_1, k_2 and P_1 for the different LacI roadblocks, and another set of k_1, k_2 and P_1 for the EcoRI Q111 roadblock (Table 1). The globally optimized values of k_1, k_2, and P_1 were used to generate CCDFs in Supplementary Figure 9 and main text Figure 5B-E.

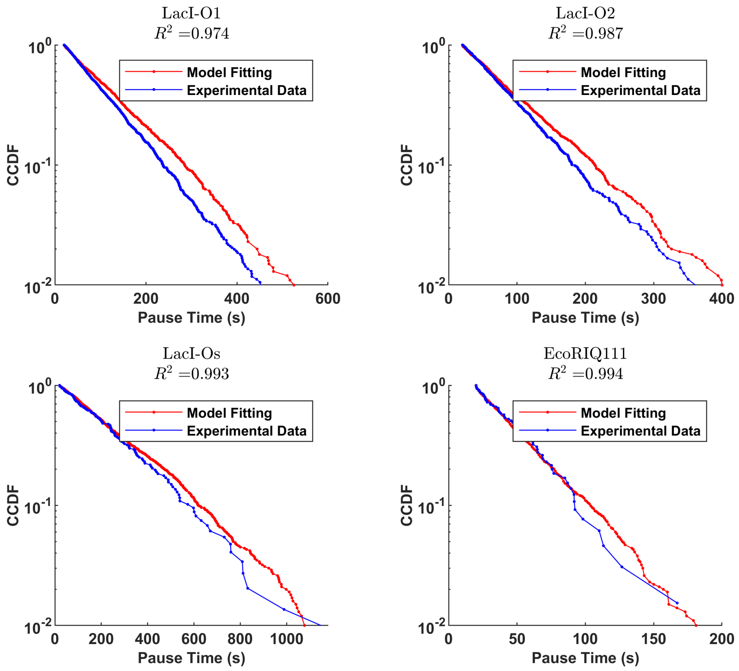

Supplementary Figure 8: Fitting k_3 using pause time CCDFs under assisting force conditions.

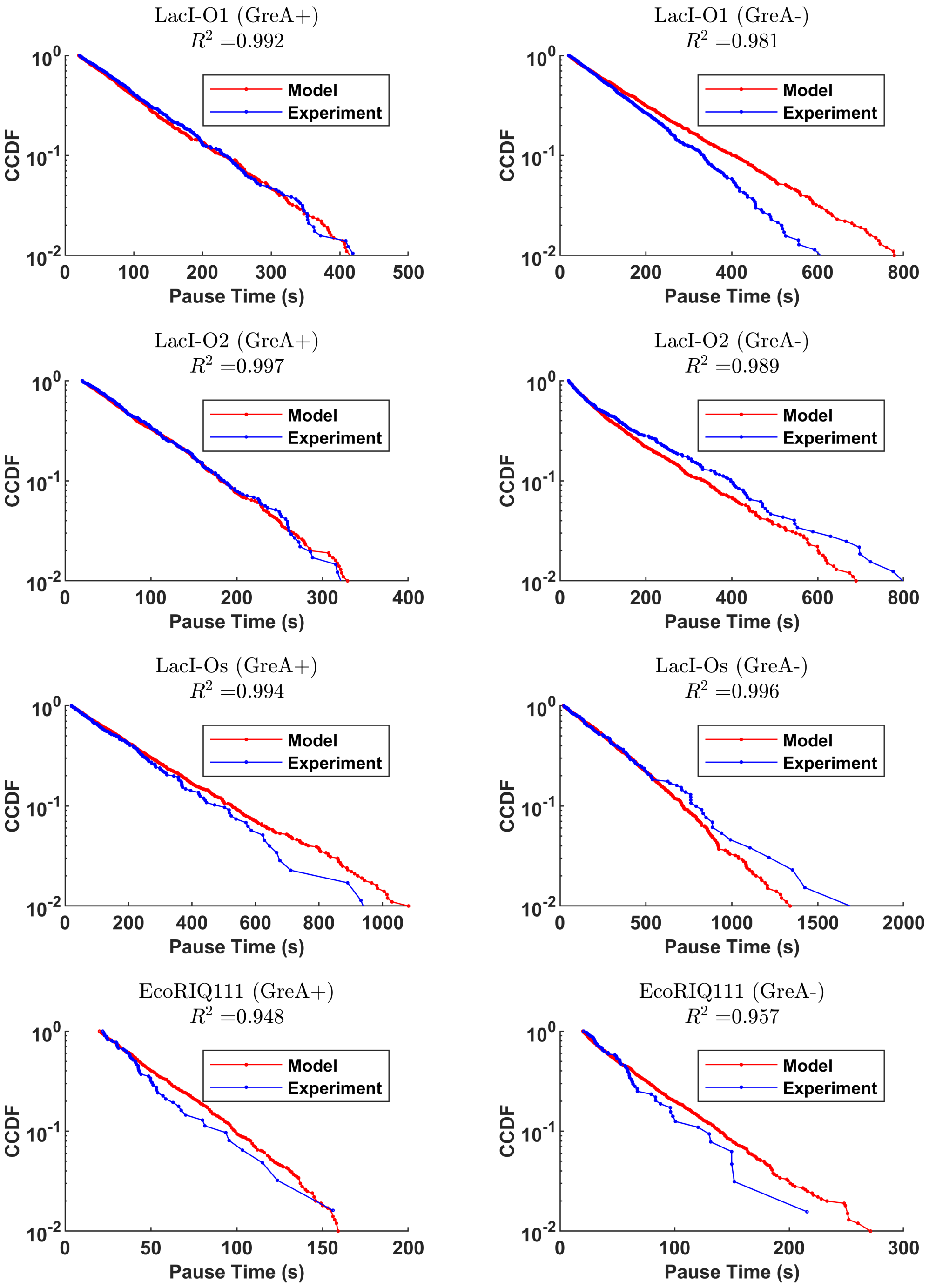

Supplementary Figure 9: Fitting k_1, k_2 and P_1 to the LacI-O1/O2/Os and EcoRI Q111 roadblock systems using pause time CCDFs under opposing force with/without GreA.

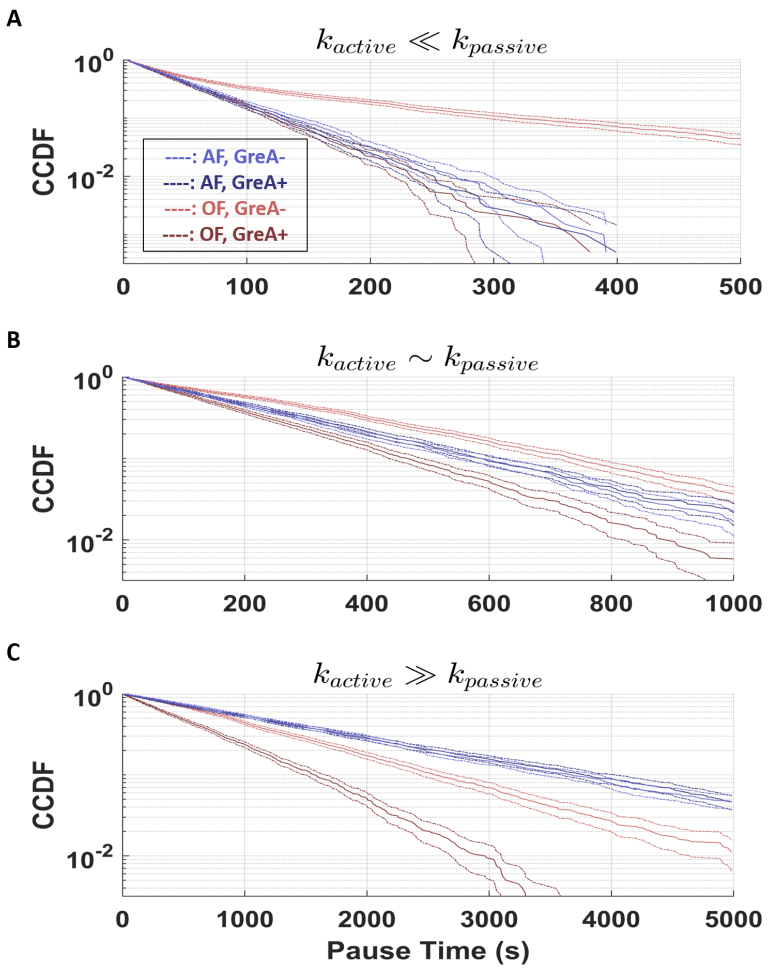

Supplementary Figure 10: **Simulations show the effects of forces and GreA in different regimes.** Rate constants used were as follows: $k_1 = 0.01s^{-1}$, $k_2 = 0.005s^{-1}$, $k_3 = 0.03s^{-1}$, $P_1 = 0.2$ (passive transit); $k_1 = 0.01s^{-1}$, $k_2 = 0.005s^{-1}$, $k_3 = 0.005s^{-1}$, $P_1 = 0.2$ (hybrid of passive and reciprocating/active transit); $k_1 = 0.01s^{-1}$, $k_2 = 0.005s^{-1}$, $k_3 = 0.001s^{-1}$, $P_1 = 0.2$ (reciprocating/active transit). The model produced CCDF showing: (**A**) $\tau_{(\text{OF,GreA-})} > \tau_{(\text{OF,GreA+})} \sim \tau_{(\text{AF,GreA-})} \sim \tau_{(\text{OF,GreA+})}$ ($k_{\text{active}} \ll k_{\text{passive}}$, passive transit); (**B**) $\tau_{(\text{OF,GreA-})} > \tau_{(\text{AF,GreA-})} \sim \tau_{(\text{AF,GreA+})} > \tau_{(\text{OF,GreA+})}$ ($k_{\text{active}} \sim k_{\text{passive}}$, hybrid of passive and reciprocating/active transit) and (**C**) $\tau_{(\text{AF,GreA-})} \sim \tau_{(\text{AF,GreA+})} > \tau_{(\text{OF,GreA-})} > \tau_{(\text{OF,GreA+})}$ ($k_{\text{active}} \gg k_{\text{passive}}$, reciprocating/active transit).

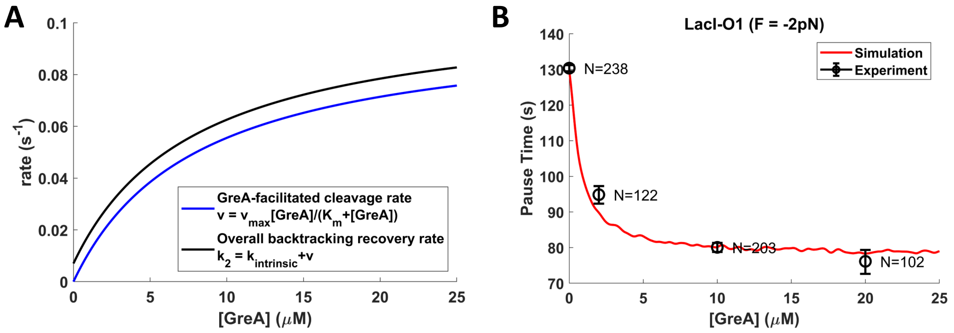

Supplementary Figure 11: Experiments with different GreA concentrations show effects on pause times similar to those of simulations. (**A**) Assuming a Michaelis-Menten relationship, the rate of GreA-induced cleavage of nascent RNA in backtracked RNAP complexes is shown in blue. The rate is estimated using parameters $K_m = 8$ μM (a reasonable guess according to the empirical results [3]) and $v_{max} = 0.1s^{-1}$. The overall backtrack recovery rate k_2 (black curve) is evaluated as the sum of the intrinsic cleavage rate (from fitting backtrack recovery rate from experimental data in [GreA] = 0 condition, see Supplementary Table 1) and the GreA-facilitated cleavage rate. (**B**) Simulation was executed by iterating Algorithm 1 in the main text to generate pause time distributions at various GreA concentrations, using the estimated k_2 values in panel (A) and fitted k_1, k_3 and P_1 values in Supplementary Table 1. The range of GreA concentration was taken as 0 – 25 μM with 0.1 μM increment, and Algorithm 1 was repeated 10,000 times at each GreA concentration. The red curve shows the characteristic lifetime of simulated pause time distributions as a function of GreA concentration. Exponential fits ± 90% confidence interval of pause times collected in experiments at 0, 2, 10, and 20 μM GreA concentrations (black) agree with the simulation results.

Supplementary Table 1: Kinetic parameters generated by fitting our model to the experimental pause time distributions. (* k_3 values postulated from pause time CCDFs, rather than model fitting, due to the large fraction of indefinite stalls in the experimental data set.)

	k_{passive} (s^{-1})	k_1 (s^{-1})	k_2 (s^{-1})	P_1	k_{active} (s^{-1})
LacI-O2	0.0119	0.0087	0.0070	0.2549	0.0038
LacI-O1	0.0094	0.0087	0.0070	0.2549	0.0038
LacI-Os (Indefinite stalls excluded)	0.0034	0.0087	0.0070	0.2549	0.0038
EcoRI(150mM[KGlu]) (Indefinite stalls excluded)	0.0331	0.0103	0.0158	0.1267	0.0062
LacI-Os	0.001*	0.0087	0.0070	0.2549	0.0038
EcoRI(150mM[KGlu])	0.01*	0.0103	0.0158	0.1267	0.0062

Supplementary References

[1] Qian, J., Dunlap, D., Finzi, L.: Thermodynamic model of bacterial transcription. Phys. Rev. E **106**, 044406 (2022) https://doi.org/10.1103/PhysRevE.106.044406

[2] Little, M.A., Jones, N.S.: Sparse bayesian step-filtering for high-throughput analysis of molecular machine dynamics. In: 2010 IEEE International Conference on Acoustics, Speech and Signal Processing, pp. 4162–4165 (2010). https://doi.org/10.1109/ICASSP.2010.5495722

[3] Maddalena, L.L.d., Niederholtmeyer, H., Turtola, M., Swank, Z.N., Belogurov, G.A., Maerkl, S.J.: Grea and greb enhance expression of escherichia coli rna polymerase promoters in a reconstituted transcription–translation system. ACS Synthetic Biology **5**(9), 929–935 (2016) https://doi.org/10.1021/acssynbio.6b00017
