# Supplementary material for: Reciprocating RNA Polymerase batters through roadblocks
Source: Nat Commun. 2024 Apr 12;15:3193. doi: 10.1038/s41467-024-47531-x (PMC11014978; doi:10.1038/s41467-024-47531-x)
Supplement: Supplementary file 3 — Description of additional supplementary files [file 41467_2024_47531_MOESM3_ESM.pdf]

Description of Additional Supplementary Files

File Name: Supplementary Data 1

Description: This is a Genbank format file containing the sequence of the pWX_12_400 plasmid1 diagrammed in the Supplementary Information. Annotations indicate the type and positions of the promoter and terminator, the roadblock binding sites, the primer pairs used to amplify opposing or assisting transcription templates, and the antibiotic selection elements.

File Name: Supplementary Data 2

Description: This is a Genbank format file containing the sequence of the pDM_N1_400 plasmid2 diagrammed in the Supplementary Information. Annotations indicate the type and positions of the promoter and terminator, the roadblock binding site, the primer pairs used to amplify opposing or assisting transcription templates, and the antibiotic selection elements.

File Name: Supplementary Data 3

Description: This is a Genbank format file containing the sequence of the pZV_NI_400 plasmid diagrammed in the Supplementary Information. Annotations indicate the type and positions of the promoter and terminator, the roadblock binding site, the primer pairs used to amplify opposing or assisting force transcription templates, and the antibiotic selection elements.

File Name: Supplementary Data 4

Description: This is a Genbank format file containing the sequence of the pDM_E1_400 plasmid diagrammed in the Supplementary Information. Annotations indicate the type and positions of the promoter and terminator, the roadblock binding site, the primer pairs used to amplify opposing or assisting transcription templates, and the antibiotic selection elements.

File Name: Supplementary Data 5

Description: This is a PDF file containing maps of the plasmid used with annotations indicating the type and positions of the promoter and terminator, the roadblock binding site, the primer pairs used to amplify opposing or assisting transcription templates, and the antibiotic selection elements.
