# Supplementary figures and images for: Reciprocating RNA Polymerase batters through roadblocks
Source: Nat Commun. 2024 Apr 12;15:3193. doi: 10.1038/s41467-024-47531-x (PMC11014978; doi:10.1038/s41467-024-47531-x)

# Plasmid maps

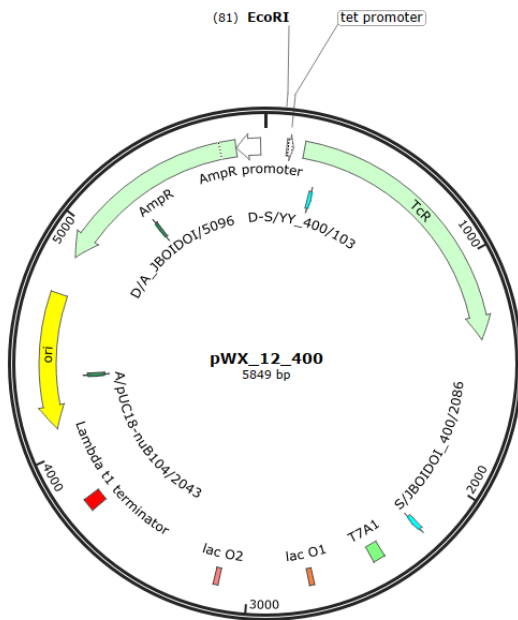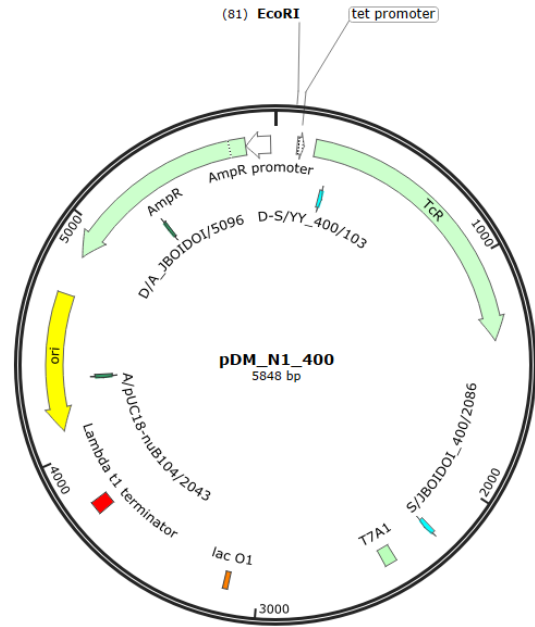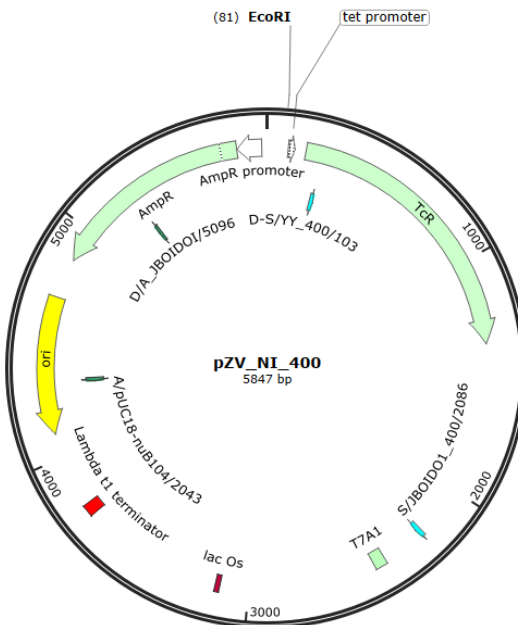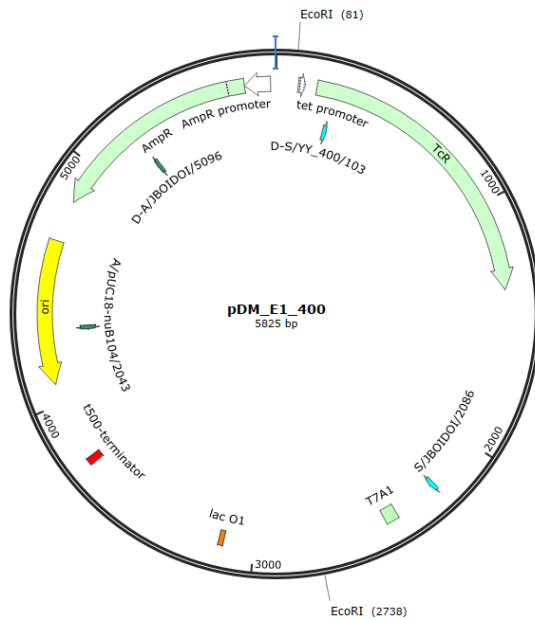

Supplement: Supplementary file 8 — Supplementary Data 5 [file 41467_2024_47531_MOESM8_ESM.pdf]
