# Supplementary material for: Reciprocating RNA Polymerase batters through roadblocks
Source: Nat Commun. 2024 Apr 12;15:3193. doi: 10.1038/s41467-024-47531-x (PMC11014978; doi:10.1038/s41467-024-47531-x)
Supplement: Supplementary file 9 — Reporting Summary [file 41467_2024_47531_MOESM9_ESM.pdf]

nature portfolio

Corresponding author(s): Laura Finzi

Last updated by author(s): Mar 26, 2024

Reporting Summary

Nature Portfolio wishes to improve the reproducibility of the work that we publish. This form provides structure for consistency and transparency in reporting. For further information on Nature Portfolio policies, see our Editorial Policies and the Editorial Policy Checklist.

Statistics

For all statistical analyses, confirm that the following items are present in the figure legend, table legend, main text, or Methods section.

n/a	Confirmed	
☐	☒	The exact sample size (*n*) for each experimental group/condition, given as a discrete number and unit of measurement
☐	☒	A statement on whether measurements were taken from distinct samples or whether the same sample was measured repeatedly
☐	☒	The statistical test(s) used AND whether they are one- or two-sided<br>*Only common tests should be described solely by name; describe more complex techniques in the Methods section.*
☐	☒	A description of all covariates tested
☐	☒	A description of any assumptions or corrections, such as tests of normality and adjustment for multiple comparisons
☐	☒	A full description of the statistical parameters including central tendency (e.g. means) or other basic estimates (e.g. regression coefficient) AND variation (e.g. standard deviation) or associated estimates of uncertainty (e.g. confidence intervals)
☐	☒	For null hypothesis testing, the test statistic (e.g. *F*, *t*, *r*) with confidence intervals, effect sizes, degrees of freedom and *P* value noted<br>*Give P values as exact values whenever suitable.*
☐	☒	For Bayesian analysis, information on the choice of priors and Markov chain Monte Carlo settings
☒	☐	For hierarchical and complex designs, identification of the appropriate level for tests and full reporting of outcomes
☒	☐	Estimates of effect sizes (e.g. Cohen's *d*, Pearson's *r*), indicating how they were calculated

Our web collection on statistics for biologists contains articles on many of the points above.

Software and code

Policy information about availability of computer code

Data collection	We used a customized Matlab based Magnetic Tweezers software to collect original data. The original data were stored in a binary format and then converted into Matlab.mat file for further analysis. All code related to data collection with our magnetic tweezer microscope is available upon request.
Data analysis	All codes were written in Matlab R2023b, including the statistical analysis toolbox and other customized codes. We have made available any code related to data analysis on Figshare (DOI https://doi.org/10.6084/m9.figshare.24782556), together with raw data sets.

For manuscripts utilizing custom algorithms or software that are central to the research but not yet described in published literature, software must be made available to editors and reviewers. We strongly encourage code deposition in a community repository (e.g. GitHub). See the Nature Portfolio guidelines for submitting code & software for further information.

Data

Policy information about availability of data

All manuscripts must include a data availability statement. This statement should provide the following information, where applicable:

- Accession codes, unique identifiers, or web links for publicly available datasets
- A description of any restrictions on data availability
- For clinical datasets or third party data, please ensure that the statement adheres to our policy

The data underlying the findings of this study are available in ``figshare'' at DOI https://doi.org/10.6084/m9.figshare.24782556. Source data are provided with this paper.

Research involving human participants, their data, or biological material

Policy information about studies with human participants or human data. See also policy information about sex, gender (identity/presentation), and sexual orientation and race, ethnicity and racism.

Reporting on sex and gender	N/A
Reporting on race, ethnicity, or other socially relevant groupings	N/A
Population characteristics	N/A
Recruitment	N/A
Ethics oversight	N/A

Note that full information on the approval of the study protocol must also be provided in the manuscript.

Field-specific reporting

Please select the one below that is the best fit for your research. If you are not sure, read the appropriate sections before making your selection.

☒ Life sciences ☐ Behavioural & social sciences ☐ Ecological, evolutionary & environmental sciences

For a reference copy of the document with all sections, see nature.com/documents/nr-reporting-summary-flat.pdf

Life sciences study design

All studies must disclose on these points even when the disclosure is negative.

Sample size	We collected about 10-20 transcription events per micro-chamber and aimed at a lower limit of about 50 transcription events in each condition to obtain a reliable distribution of data points. However, we collected over 200 transcription events in each experimental condition tested which were more than sufficient to support our findings.
Data exclusions	Pauses shorter than 20 s were excluded from further analysis as described in the manuscript
Replication	Given the variability among individual protein and DNA molecules, it is difficult to perform technical replicates of our experiment, since individual transcription rates and pausing events depend on the actual single molecules involved. In other words, it is exceedingly difficult to select protein activity and DNA structure, the “biological variability”, down to the single molecule level to be able to separate technical and biological variabilities. In addition, the number of molecules recorded in each experiment is insufficient to perform exponential fits of the data. Therefore, we pooled the data from multiple experiments to perform the fitting. Including all micro-chambers examined on different days, there were 6308 distinct DNA molecules that exhibited transcription and termination events at expected tether lengths. Following the exponential fitting analyses significant pausing was observed only at lac repressor binding sites when LacI was added to the sample.
Randomization	At the beginning of an experiment, a randomly selected view with approximately twenty or more particles exhibiting movement, which appear to be tethered by DNA and not stuck on the surface, were selected for tracking. Then NTPs were added, and all records displaying progressive tether length shortening were included in subsequent analyses. A threshold of 20 seconds for roadblock-induced pausing was set based on previous work in the literature in which transcription elongation complexes were observed to pause as long as 10 seconds. Pauses shorter than this 20 second threshold were excluded using an automated routine. Randomization was inherent because DNA tethers with active transcription complexes could not be predicted a priori.
Blinding	Automation of the pause analysis ensured that the pauses detected in the various conditions were not subject to experimenter bias.

Reporting for specific materials, systems and methods

We require information from authors about some types of materials, experimental systems and methods used in many studies. Here, indicate whether each material, system or method listed is relevant to your study. If you are not sure if a list item applies to your research, read the appropriate section before selecting a response.

Materials & experimental systems

n/a	Involved in the study	
☐	☒	Antibodies
☒	☐	Eukaryotic cell lines
☒	☐	Palaeontology and archaeology
☒	☐	Animals and other organisms
☒	☐	Clinical data
☒	☐	Dual use research of concern
☒	☐	Plants

Methods

n/a	Involved in the study	
☒	☐	ChIP-seq
☒	☐	Flow cytometry
☒	☐	MRI-based neuroimaging

Antibodies

Antibodies used	Anti-digoxigenin, cat#: 11333089001; lot # 66890900 (Meets Roche specifications as per their statement); Concentration: 8 micrograms/ml.
Validation	lot # 66890900 (Meets Roche specifications as per their statement)
